# Consumer Awareness of the Degree of Industrial Food Processing and the Association with Healthiness—A Pilot Study

**DOI:** 10.3390/nu14204438

**Published:** 2022-10-21

**Authors:** Dieuwerke Bolhuis, Ana Carolina Mosca, Nicoletta Pellegrini

**Affiliations:** 1 Food Quality and Design, Wageningen University and Research, P.O. Box 17, 6700 AA Wageningen, The Netherlands; 2 Food and Drug Department, University of Parma, 43124 Parma, Italy; 3 Department of Agricultural, Food, Environmental and Animal Sciences, University of Udine, 3310 Udine, Italy

**Keywords:** industrial food processing, ultraprocessed foods, NOVA classification, Nutri-Score, healthiness perception, consumer awareness

## Abstract

Consumption of ultraprocessed foods (UPFs) has been associated with lower diet quality, obesity, and adverse health effects. Not much is known about how consumers evaluate the degree of processing of a food product and how they relate this to healthiness. An online questionnaire was completed by a total of 277 Dutch, 204 Italian, and 181 Brazilian consumers. Consumers were aged 18–65 year, mean 38 ± 13 year, 31% were males, and 71% were highly educated. Pictures of several common food products were evaluated on the degree of industrial processing and healthiness. Thirteen food categories were included, each including one minimally processed food (MPF), one High NS_UPF (Nutri-Score A or B), and one Low NS_UPF (Nutri-Score D or E). Lastly, knowledge and attitude about UPFs were assessed. Ultraprocessing was perceived as unhealthy by the majority of consumers (Dutch, Italian: 55%; Brazilian: 75%) and contributed to weight gain according to: 38% Dutch, 51% Italian, and 70% Brazilian consumers. Low NS_UPFs were correctly rated toward “processed” and “not healthy” in all countries. High NS_UPF were rated as processed but showed large variations in healthiness scores. In conclusion, consumers rated UPFs relatively low in healthiness compared with MPFs with similar Nutri-Scores within the same food category. These preliminary findings suggest that consumers incorporate, to some extent, the degree of industrial processing while assessing the healthiness of food products.

## 1. Introduction

Recently, the industrial processing of foods and its possible adverse effects on health have been widely debated in the media and scientific literature [1,2,3,4,5,6,7]. In theory, food processing refers to any transformation from agricultural products to foods, which includes simple techniques such as cutting or heating at a household level, to highly advanced techniques such as extracting, extruding, fermenting, pressuring, and hydrogenating at the industrial level [8,9]. Industrial food processing has historically been performed to produce safe and palatable foods with an extended shelf-life [10]. Several systems have been developed that classify foods based on the degree of processing [11,12]. The NOVA classification, the most well-known classification system, introduced the term “ultraprocessed foods” (UPFs). Consumption of UPFs has been linked to overconsumption and weight gain [5,13], obesity, and chronic diseases [2,3,4,14].

The NOVA classification was developed at the University of São Paulo in Brazil [15]. This system classifies foods into four categories depending on the nature, extent, and purpose of processing. NOVA 1 consists of unprocessed foods and minimally processed foods (MPFs), including fruits, vegetables, and fresh meats. NOVA 2 consists of processed culinary ingredients, including oil, sugar, and salt. NOVA 3 consists of processed foods, including canned or bottled vegetables, fruits in syrup, and salted nuts. NOVA 4 consists of UPFs, which are defined as: “formulations of ingredients, mostly of exclusive industrial use, that result from a series of industrial processes” [1]. UPFs are basically all foods that do not fit in the other groups, from obvious unhealthy foods such as chicken nuggets, sugar-sweetened beverages, confectionery, and diet sodas, to breads, breakfast cereals, spreadable fats, flavored milks, yogurts, and many infant and toddler prepared foods [1,11]. UPFs are commonly consumed in countries worldwide, with percentages of energy intake derived from UPFs being estimated at 50–70% in the US, 50–60% in Germany and The Netherlands, ~40% in Australia, 20–30% in France, Brazil and Spain, and 10% in Italy [16,17,18,19,20,21]. The founders of the NOVA classification system advocate avoiding all UPFs, and this has influenced the dietary guidelines of many South American countries, as well as Canada, Belgium, and France [12,22].

To guide consumers in making healthy food choices, the Nutri-Score, a front-of-package (FOP) nutrition label, has been developed [23]. Several European countries have adopted the Nutri-Score, including The Netherlands, Spain, Luxemburg, Belgium, Germany, France, and Switzerland [24]. The Nutri-Score is calculated based on nutrient composition and the presence of some ingredients, such as vegetables, fruits, legumes, nuts, and olive, rapeseed, and walnut oils. The calculation is based on a sum of “unhealthy” components such as energy (kJ), total sugar (g), saturated fatty acids (g), and sodium (mg), and “healthy” components, such as fruits (%), vegetables (%), nuts (%), oils (%), fiber (g), and protein (g). The end scores are placed in categories from A (most healthy) to E (most unhealthy) [23]. To date, there is no gold standard FOP to assess the level of the healthiness of foods. However, the Nutri-Score has been demonstrated to be highly informative for guiding consumer purchases and is now widely used in several European countries.

Consumers receive conflicting and dynamic information about what is healthy from different sources, such as official nutritional recommendations, health professionals, friends and family, social media, and the press. A good understanding of how people conceptualize healthy foods and how they make their judgments about food healthiness is still lacking. These judgments are expected to be made without much deliberation and based on heuristic strategies [25,26]. Moreover, not much is known about consumers’ knowledge and opinions on industrial food processing. If consumers have a negative opinion about food processing, it might be challenging to select foods based on the degree of industrial processing [27,28,29]. Some foods have UPF status but are labeled as “healthy” according to FOP or other messages on the packaging. These conflicting messages through different sources might be confusing for consumers.

To the best of our knowledge, there is limited research into consumers’ beliefs and knowledge about food processing in relation to healthiness and their ability to judge the degree of industrial processing, especially outside South America. The aim of this pilot study was to assess consumers’ knowledge and perception of the NOVA classification and UPFs in relation to healthiness in The Netherlands, Italy, and Brazil.

## 2. Materials and Methods

### 2.1. Study Design

An online questionnaire was developed in which consumers rated the healthiness and the degree of processing of various food items. For 13 food categories, there were pictures of an MPF product, a Low NS_UPF product (according to Nutri-Score D or E), and a High NS_UPF product (according to Nutri-Score A or B). The questionnaire ended with some general questions about knowledge and attitude toward the NOVA classification and UPFs.

### 2.2. Consumers

A convenience sample of consumers aged between 18 and 65 years was recruited via the social media of researchers, colleagues, and friends (Facebook and LinkedIn), and in The Netherlands via a database of participants of consumer studies of Wageningen University and Research. Data from consumers were excluded when the questionnaire was incomplete (Netherlands, *n* = 27; Italy, *n* = 33; Brazil, *n* = 37) and when the same answer was given to all questions (Brazil, *n* = 1). In the end, data from 277 Dutch, 204 Italian, and 181 Brazilian consumers were included.

### 2.3. Food Products Selection

Food product selection was conducted via assessment of the most frequently consumed food groups in The Netherlands [30], Italy [31], and Brazil [32], according to national consumption data. For each food product, the NOVA category and the Nutri-Score were determined via Open Food Facts (https://world.openfoodfacts.org/ assessed on 20 October 2022) or, if not available, were calculated based on the composition of the products sold in The Netherlands [33]. When a food product scored a Nutri-Score of A or B, it was defined as “high NS”. When a food product scored a D or E on the Nutri-Score, the food product was defined as “low NS”.

For each of the 13 product categories, three food products were selected: a NOVA 1 or 3 (MPF) product, an “unhealthy” NOVA 4 (Low NS_UPF) product, and a “healthy” NOVA 4 (High NS_UPF) product (Table 1, see Supplementary Table S1 for product pictures). Most MPFs were healthy and had a Nutri-Score of A or B, except for cheese (Nutri-Score D). For three food product categories (yogurt, ready-to-eat, and tomato), a UPF with a Nutri-Score of C was used, as there were no unhealthy UPFs with a Nutri-Score of D or E. The pictures of food products were selected via a food-pics extended database [34,35] and via a search on the internet. All product pictures had a neutral background and were presented from the same angle.

### 2.4. Questionnaire

The questionnaire was developed using the software Qualtrics (version 2020, Qualtrics, Provo, UT, USA). Consumers did not know the exact purpose of the questionnaire. They were informed that the goal of the project was to obtain their opinion about several food products. The duration of the questionnaire was approximately 5–10 min. Per food item, consumers saw a picture of the food and rated the degree of processing and healthiness on a line scale. The line scale was anchored at the two most extreme ends. Processing was anchored at “not processed at all” and “very processed”. Healthiness was anchored at “not healthy at all” and “very healthy”. Each participant answered these questions for 15 products (i.e., five MPFs, five High NS_UPFs, and five Low NS_UPFs) randomly selected from a total of 39 food products. Rating all 39 food products was expected to result in fatigue and quitting the questionnaire without finishing.

After rating the food pictures, there were a few questions about the NOVA classification and UPFs. The first question was: “The NOVA system is used to classify food products. According to you, NOVA separates foods based on (single choice): nutritional composition, the extent of food processing, product sustainability, or I don’t know the NOVA classification system”. The second question was, “According to you, ultraprocessed foods are (more than one option can be selected): goods composed with more than 5 ingredients, food products submitted to a series of industrial processing, genetically modified products, food products that contain artificial ingredients, or I don’t know what ultraprocessed foods are”. The third question was: “In your opinion, ultraprocessed foods are”. This was rated on a line scale anchored from “not healthy at all” (1) to “very healthy” (7) on a continuous Likert scale. The fourth question was: “Ultraprocessed foods contribute to weight gain”. This was rated on a line scale anchored from “strongly disagree” to “strongly agree”. Lastly, the questionnaire contained questions about sociodemographic characteristics: age, nationality, gender, education level, and income level. The questionnaire was presented in the official language of each country. Data were collected in November and December 2020.

### 2.5. Data Analysis

Ordinal data are presented as median with interquartile range (IQR), while nominal and categorical data are presented in frequencies with percentages. To present all demographic consumer characteristics, age was divided into five categories: 18–25 (*n* = 173), 26–35 (*n* = 134), 36–45 (*n* = 142), 46–55 (*n* = 129), and 56–65 (*n* = 84) years, and percentages were calculated for gender, education level, and income level. Pearson’s chi-square tests were performed to assess whether categorical data were differently distributed between countries.

**Figure 1:**
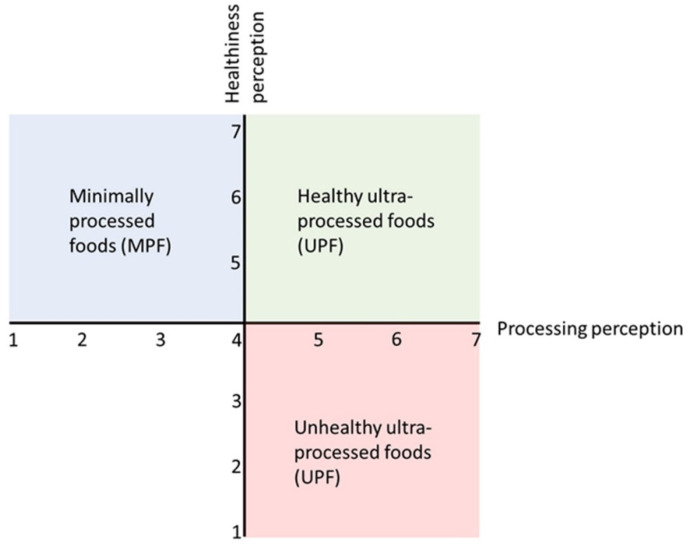
Expected distribution of food products based on the degree of healthiness and processing.

Regarding the questions about the food product pictures, the two extreme points of the processing and healthiness scales were converted into a 7-point Likert scale, with 1, not processed/healthy at all; 2, not healthy/processed; 3, slightly not healthy/processed; 4, neither; 5, slightly healthy/processed; 6, healthy/processed; and 7, very healthy/processed. The calculated distribution of food products based on the differences in healthiness and processing is shown in Figure 1. MPFs were expected to score from 1 to 4 on processing and 4 to 7 on healthiness (blue area). High NS_UPFs were expected to score from 4 to 7 on processing and 4 to 7 on healthiness (green area). Low NS_UPFs were expected to score from 4 to 7 on processing and 1 to 4 on healthiness (red area).

For each country, a scatterplot containing all food products was made. A Spearman correlation (*r_s_*) was performed to study the strength of the relationship between processing and healthiness. Pearson’s chi-square tests were used to compare frequencies, and Kruskal–Wallis tests were used to compare ordinal data. Statistical analyses were performed with the software SPSS statistics version 26 (IBM SPSS Statistics, IBM corp., Chicago, IL, USA). A *p*-value of ≤0.05 was considered significant.

## 3. Results

### 3.1. Consumer Characteristics

Sociodemographic characteristics of 277 Dutch, 181 Brazilian, and 204 Italian consumers are presented in Table 2. The median age of the study population for The Netherlands, Italy, and Brazil was 34, 41, and 38 years, respectively. In all populations, there were more female responders, and most consumers had a high educational level. Both Dutch and Brazilian consumers were mostly higher educated, whereas Italian consumers were more frequently moderately educated (*p* < 0.001). More students participated among Dutch consumers compared to the other countries. Italian and Brazilian consumers more frequently had a moderate income level, whereas Dutch consumers more frequently had either a low or high income (*p* < 0.001).

### 3.2. Knowledge of and Attitude toward NOVA Classification and UPFs

**Figure 2:**
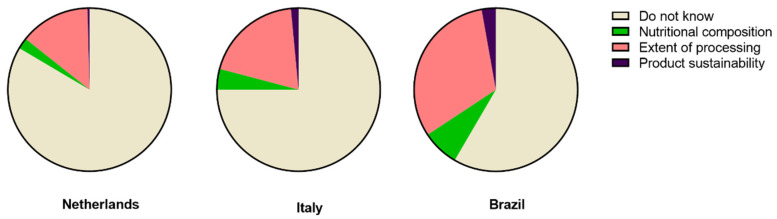
Multiple choice answers (%) to the question: “According to you, NOVA separates foods based on: …” by Dutch (*n* = 277), Italian (*n* = 204), and Brazilian (*n* = 181).

When consumers were asked whether they knew the NOVA classification, 84% of Dutch consumers indicated that they did not know the NOVA classification compared with 75% of Italian consumers and 58% of Brazilian consumers (Figure 2). The distribution of answers was different between the three countries (*p* < 0.001). Among the Brazilian consumers, 32% answered that the NOVA classification was related to the extent of processing, while just 14% of the Dutch and 19% of the Italian consumers gave this correct answer (*p* < 0.001).

**Figure 3:**
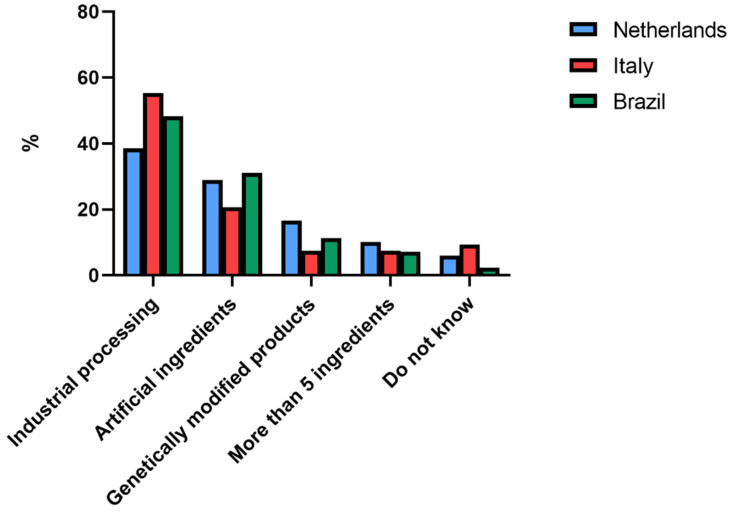
Answers (%) to the multiple choice question: “According to you, ultraprocessed foods are (more than one option can be selected): …” by Dutch (*n* = 277), Italian (*n* = 204), and Brazilian (*n* = 181) consumers.

The knowledge of Dutch, Italian, and Brazilian consumers about the term UPF is given in Figure 3. In all three countries, consumers most frequently answered that UPFs were “food products submitted to a series of industrial processing”, followed by the answer “food products that contain artificial ingredients”. The term UPF was more familiar than the NOVA classification, with just 5.9% of Dutch, 9.2% of Italian, and 2.3% of Brazilian consumers not familiar with the term UPF. Distributions were not significantly different between countries.

**Figure 4:**
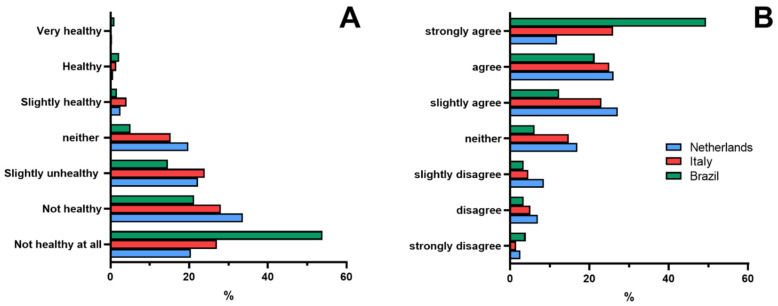
Healthiness perception of UPFs of Dutch (*n* = 277), Italian (*n* = 204), and Brazilian (*n* = 181) consumers (**A**). Agreement with the statement that UPF contributes to weight gain (**B**).

Furthermore, UPFs were rated as unhealthy by 55% of Dutch and Italian consumers compared with 75% of Brazilian consumers (Figure 4A). Italy and The Netherlands had a similar distribution (*p* = 1.0), while the distribution of answers in Brazil differed from Italy and The Netherlands (*p* < 0.001). Figure 4B shows the opinion on whether UPF contributes to weight gain; 70% of Brazilians agreed with this statement compared with 38% of Dutch consumers and 51% of Italian consumers (*p* < 0.001).

### 3.3. Processing and Healthiness Ratings of Various Food Products

**Figure 5:**
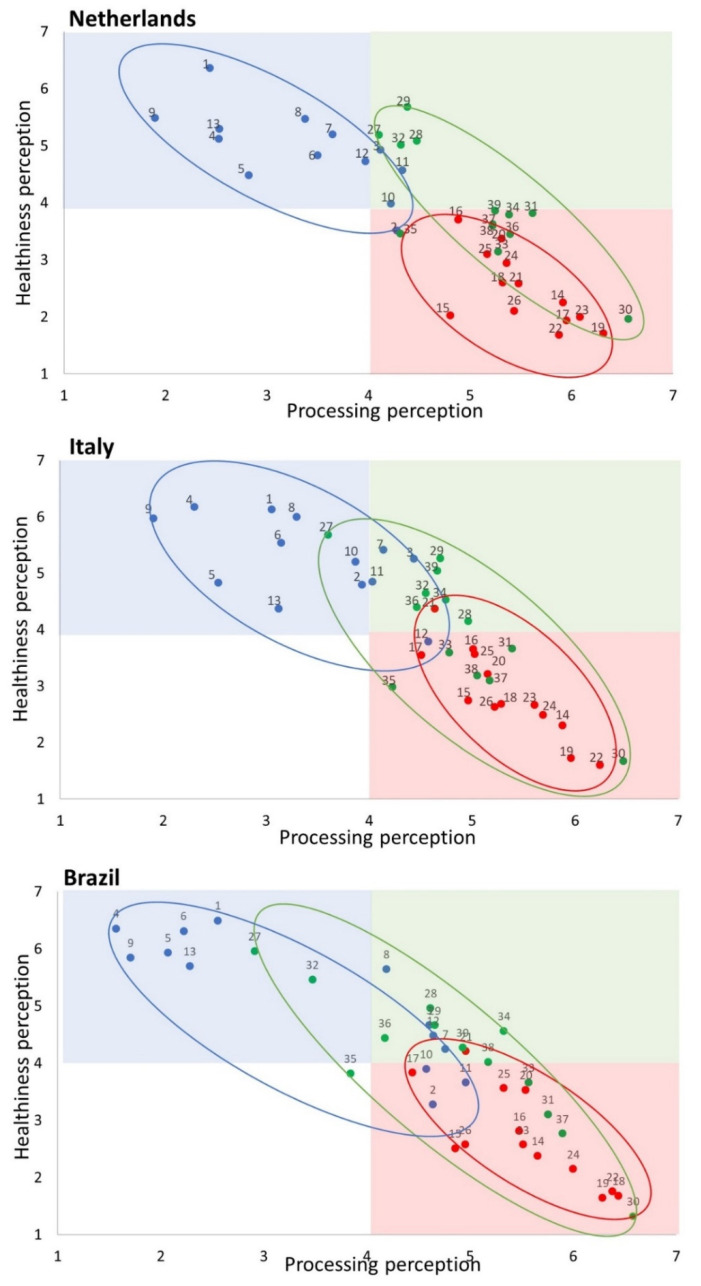
Median values of ratings of degree of processing and healthiness of 39 food products divided into three categories (MPF (blue dots), High NS_UPF (green dots), and Low NS_UPF (red dots): 1, oatmeal; 2, French bread; 3, rice waffle; 4, orange juice; 5, steak; 6, chicken filet; 7, semiskimmed milk; 8, plain yogurt; 9, boiled potato; 10, fusilli; 11, canned tomato; 12, young matured cheese; 13, peanuts; 14, chocolate cereals; 15, croissant; 16, wheat cookie; 17, lemonade; 18, sausage; 19, chicken nuggets; 20, fruit milk; 21, stracciatella; 22, paprika chips; 23, pasta ham and cheese; 24, tomato ketchup; 25, cream cheese; 26, chocolate peanuts; 27, granola; 28, packaged bread; 29, grain cracker; 30, cola light; 31, vegetable burger; 32, tofu; 33, chocolate milk without added sugars; 34, fruit yogurt; 35, oven fries; 36, pasta pesto with vegetables; 37, reduced sugar and salt ketchup; 38, cottage cheese; 39, oatmeal bar.

Figure 5 shows the median ratings of healthiness and the degree of processing of the 39 food products (see Table 1). In all countries, there was a strong negative correlation between processing and healthiness perception: The Netherlands, *r_s_* = −0.81; Italy, *r_s_* = 0.75; Brazil, *r_s_* = −0.90; all *p* < 0.001. It was expected that the MPFs would be placed in the blue space (lower degree of processing and higher healthiness), the High NS_UPFs in the green space (higher degree of processing and higher in healthiness), and the Low NS_UPFs in the red space (higher degree of processing and lower in healthiness). The circles (blue including MPFs, green including High NS_UPFs, red including Low NS_UPFs) show that consumers did not always rate the food products in line with the actual degree of processing according to NOVA or the degree of healthiness according to the Nutri-Score. Especially the High NS_UPFs (green) were, in many cases, rated toward lower healthiness (red). MPFs were mostly rated as healthy and having a lower degree of processing, with some exceptions in Brazilian and Italian consumers on processing. All Low NS_UPFs were “correctly” rated in the red area, except for full-fat stracciatella yogurt in Italian and Brazilian consumers.

#### 3.3.1. MPFs

According to Dutch and Italian consumers, most MPFs scored high on healthiness and low on processing. Four MPFs scored slightly higher on processing than expected, including rice waffle and canned tomato in both countries, French bread and fusilli in The Netherlands, and semiskimmed milk and young matured cheese in Italy. Brazilian consumers perceived seven MPFs (French bread, rice waffle, semiskimmed milk, plain yogurt, fusilli, canned tomato, and young matured cheese) as more processed than expected, of which three products (French bread, fusilli, and canned tomato) were perceived as slightly unhealthy. In general, in all countries, some MPFs were perceived as slightly more processed than expected.

#### 3.3.2. Low_NS and High NS_UPFs

Dutch consumers rated all UPFs as higher in processing. Granola (no. 27) in Italy and granola, tofu (no. 32), and oven fries (no. 35) in Brazil were rated as less processed than expected. In all countries, almost all 13 Low NS_UPFs were rated as expected—high on processing and low on healthiness—except for full-fat stracciatella yogurt, which was perceived as slightly healthy by Brazilian and Italian consumers.

In all countries, the 13 High NS_UPFs were more spread across processing and healthiness perception compared with MPFs and unhealthy UPFs. Dutch consumers perceived four High NS_UPFs within the expected range, namely granola (no. 27), packaged bread (no. 28), grain cracker (no. 29), and tofu (no. 32). Six High NS_UPFs (cola light (no. 30), chocolate milk without added sugars (no. 33), oven fries (no. 35), pasta pesto with vegetables (no. 36), reduced sugar and salt ketchup (no. 37), and cottage cheese (no. 38)) were perceived as less healthy than the actual Nutri-Score classification, and three products were rated as neutral in healthiness (vegetable burger (no. 31), fruit yogurt (no. 34), and oatmeal bar (no. 39)). According to Brazilian and Italian consumers, six High NS_UPFs (packaged bread (no. 28), grain cracker (no. 29), fruit yogurt (no. 34), pasta pesto with vegetables (no. 36), oatmeal bar (no. 39), and tofu (no. 32)) were perceived within the expected range of healthiness. Five High NS_UPFs were rated toward unhealthy in both countries (cola light (no. 30), vegetable burger (no. 31), chocolate milk without added sugars (no. 33), oven fries (no. 35), reduced sugar and salt ketchup (no. 37)) and cottage cheese (no. 38) in Italy.

#### 3.3.3. Comparison of MPFs and UPFs within Food Product Categories

In all countries, when comparing the three products within a food category, the High NS_UPFs were generally perceived as less healthy and more processed compared with the corresponding MPFs (Supplementary Tables S2 and S3 and Figure 5: the blue dots (MPFs) are generally rated higher in healthiness compared with the green dots (High NS-UPFs), with a few exceptions. In the categories cereals, beverages, meat, chicken, milk, yogurt, potatoes, ready-to-eat, tomato, and cheese, MPFs were rated higher in healthiness than High NS_UPFs. However, in the bread category, in Brazil and The Netherlands, French bread (MPF) was perceived as less healthy than packaged bread (High NS_UPF). Furthermore, grain cracker (High NS_UPF) was perceived as healthier than rice waffle (MPF) in The Netherlands.

In all countries, within each food category, almost all High NS_UPFs were perceived as healthier and less processed than the corresponding Low NS_UPFs (Supplementary Tables S2 and S3), with a few exceptions listed below. In all countries, in the beverage category, cola light (High NS_UPF) was perceived as less healthy than lemonade (Low NS_UPF). Furthermore, in the milk and cheese categories, the High NS_UPF was not rated differently in healthiness perception compared with the Low NS_UPF.

Regarding perceived food processing, the MPFs were generally rated lower in processing compared with both UPFs groups (Supplementary Table S3)). Interestingly, Low NS_UPFs were perceived as more processed compared with High NS_UPFs in five categories: cereals, biscuits, chicken, potatoes, and ready-to-eat. Both UPF groups were perceived as equally processed for bread, meat, milk, yogurt, tomato, cheese, and peanuts categories. Cola light (High NS_UPF) was rated as more processed than lemonade (Low NS_UPF).

## 4. Discussion

To the best of our knowledge, there are no studies that have evaluated consumers’ attitudes toward industrial food processing in Europe, whereas some consumer studies have been executed in South America [27,28,29]. This pilot study showed that most consumers did not know the NOVA classification but were more familiar with the term UPF. Most consumers evaluated UPF as “unhealthy”, and a substantial part of consumers agreed that the consumption of UPFs contributes to weight gain. Evaluation of the food pictures showed that consumers easily rated the Low NS_UPFs as “unhealthy” and “very processed” but showed more variation in judging MPFs and High NS_UPFs based on healthiness and processing.

The negative associations between UPF consumption and healthiness were expected due to media releases and health recommendations against the consumption of UPFs. Other studies also showed that industrial food processing was regarded as a negative contributor to healthiness in Uruguay, Argentina, Ecuador, Canada, and the US [27,29,36,37]. Consumers believe that “homemade” and “natural” foods are healthier than industrially processed foods [38,39,40]. There is a lack of trust in the food industry, with consumers being afraid of possible contaminants or chemical residues that might result from the way food is produced, formulated, and processed [10]. This may explain why many of the High NS_UPFs were perceived as (slightly) unhealthy and less healthy compared with the MPFs, despite a similar Nutri-Score categorization. This may reflect the belief that industrial food processing is bad for health and that minimally processed foods are always the healthier choice. Moreover, popular media and documentaries suggest that the food industry makes foods “hyperpalatable,” which means that the food has been made so delicious that it stimulates overconsumption [41]. Consequently, consumers have a negative attitude toward food processing and associate this with weight gain.

Consumers showed a wide variation in assessing the healthiness of especially the High NS_UPF group. In sum, nine High NS_UPFs in The Netherlands, five High NS_UPFs in Brazil, and six High NS_UPFs in Italy were perceived as slightly unhealthy or unhealthy. This may reflect the consumers’ confusion about various food classification systems and contradicting health messages. Another explanation in the present study design may be that consumers evaluated the product without packaging. We aimed to assess the consumer perception of plain products in three different countries. However, the healthiness perception of foods could be influenced by the packaging. Packaging shows the brand, FOP labels, health claims, origin of the product, and the ingredients, which all could be used as health indicators for the consumer [27,42,43]. Moreover, the shape, size, and pictures that are shown on the packaging material influence consumers’ healthiness perception [38,44]. These simple cues are especially used by consumers when it is not directly clear whether a product is healthy or not [27].

The present study showed that Brazilian consumers in this pilot were more familiar with the NOVA classification and had a stronger negative opinion about the healthiness of UPFs compared with Dutch and Italian consumers. However, the study sample size was small; thus, these results should be carefully interpreted. Nevertheless, the different attitudes of consumers to UPFs between the three countries may be the result of different dietary guidelines. The avoidance of UPFs is incorporated into the Brazilian dietary guideline, with a general recommendation: “Always prefer natural or minimally processed foods and freshly made dishes and meals to ultra-processed foods” (Ministry of Health of Brazil, 2014). Dietary guidelines in The Netherlands and Italy are both based on food categories, the Wheel of Five in The Netherlands [45], and the modern Mediterranean diet pyramid in Italy [46], without any reference to industrial processing or UPFs. However, Brazilian responders were not better at distinguishing the level of processing between MPF and UPF foods even though they have more information on UPFs from the dietary guidelines. Larger consumer studies are needed to confirm these assumptions.

Consumers were, in general, able to rate UPFs as processed, but some MPFs were rated more processed than expected, especially by Brazilian and Italian consumers. The increased awareness of Brazilian consumers regarding UPFs may have resulted in this overestimation of the level of food processing of seven food products. However, this assumption should be interpreted carefully due to the relatively low study sample size. Dairy products such as plain yogurt, skimmed milk, and matured cheese were rated as high in processing in Brazil and Italy, in line with Aguirre et al. [29], who found that milk was misclassified as UPFs by consumers in Argentina and Ecuador. Consumers in a focus group study indicated that the evaluation of industrial processing requires time and effort, and, instead, they often use easier cues such as brand, FOP labels, and design of the packaging [27]. Not only consumers but also food and nutrition experts showed low consistency in classifying foods in the NOVA groups, both with and without information on ingredients [47]. In contrast, another study that included 64 dieticians in Canada showed high concordance in processing scores and the NOVA classification, which could be the result of national advice to avoid UPFs in the dietary recommendations of Canada [36]. However, the risk of misclassification may lead to negative dietary consequences; for example, perceiving nutritious dairy products as highly processed may result in impaired diet quality.

Besides the risk of misclassifications, there is a scientific debate about whether the degree of industrial processing as such is a valid tool to judge the healthiness of foods [6,7,11,48]. If the degree of processing has negative health consequences, then underlying biological mechanisms should explain this relationship. Despite numerous association studies showing adverse health outcomes related to UPF consumption [2,3,4,13,14], causal mechanisms are not clear yet, although some suggestions have been proposed [49]. The NOVA classification uses a mix of technological dimensions and formulation considerations to define UPFs, such as the use of additives or the number of ingredients, which makes it difficult to explain adverse health aspects. There is a need to better inform the consumer about the processing steps needed and explain why certain ingredients are needed to improve food quality or safety. Consumers should also be aware that food processing could be used to increase the health status of a product, such as by nutritional fortifications or by designing foods in a way that prevents overconsumption [50]. It may help to give consumers more transparency on food formulation and food processing to restore their trust in the food industry [10]. The Nutri-Score uses nutrient composition to judge the healthiness of food. There is growing evidence that the healthiness of food is not only determined by the sum of its nutrients but also depends on the food form and its microstructure [50,51] and possibly the use of non-nutritive additions in food processing [52]. To date, there is no gold standard to judge the healthiness of foods.

The current study has several strengths. First of all, the products used in the questionnaire were divided into 13 frequently consumed product categories in the three countries, and each category had an MPF, a High NS_UPF, and a Low NS_UPF version of the product. Second, the questionnaire was the same in all three countries, with the same concepts, data collection method, and equivalent terminologies being used for the three translation versions, which increased the comparability of the results between countries. Third, all product pictures had the same neutral background and were presented from the same angle. Furthermore, most research about consumers’ perception of UPFs has been executed in South America, with a lack of information about consumer opinion and perception in Europe or other continents or countries.

However, the findings of this study were limited by the small size of the study population and its composition, which does not represent the population of the three countries. For all three countries, consumers were mostly highly educated, consisted of more females, and certain age groups were overrepresented. Furthermore, the sociodemographic characteristics of the study population were not completely comparable, which is often an issue in cross-cultural research [28]. Therefore, any differences found between countries should be taken with caution since they might be due to differences in sociodemographic characteristics. Therefore, the present findings should be confirmed in a larger study in which a more representative general population should be studied. Moreover, the NOVA category and Nutri-Score of food products were calculated based on the composition of products sold in The Netherlands; the composition of the products might be slightly different in Brazil and Italy.

As far as we know, there are limited data on the potential association between the degree of food processing and consumer healthiness perception. The findings from this pilot study demonstrate that consumers in all three countries had negative opinions about the healthiness of UPFs, with the most frequent negative responses in Brazil. Consumers clearly rated low Nutri-Score UPFs as high in processing and unhealthy. The food products that were classified as high Nutri-Score UPFs were mostly rated as higher in processing but showed large variations in perception of healthiness. This may represent the confusion of the consumer due to contradicting health messages. Education and transparency about nutrition, ingredients, and food processing may help to guide healthy food choices. In general, consumers rate UPFs relatively low in healthiness compared with MPFs with similar Nutri-Scores within the same food category, which may indicate that consumers use the degree of processing as a tool to determine healthiness. More data need to be collected to verify these outcomes.

## Figures and Tables

**Table 1:** All food categories and products grouped by degree of processing (NOVA) and healthiness (Nutri-Score).

Product Category	MPFs (Nutri-Score)	No*	Low NS_UPFs (Nutri-Score)	No*	High NS_UPFs (Nutri-Score)	No*
Cereals	Oatmeal (A)	1	Chocolate cereals (D)	14	Granola (A)	27
Bread	French bread (B)	2	Croissant (D)	15	Packaged bread with seeds (A)	28
Biscuits	Rice waffle (A)	3	Wheat cookie (E)	16	Grain cracker with seeds (B)	29
Beverages	Fresh orange juice (B)	4	Sparkling lemonade (D)	17	Cola light (B)	30
Meat	Steak (A)	5	Sausage (D)	18	Vegetable burger (A)	31
Chicken	Chicken filet (A)	6	Chicken nuggets (D)	19	Tofu (A)	32
Milk	Pasteurized semiskimmed milk (A)	7	Fruit flavored milk (E)	20	Semiskimmed chocolate milk without added sugars (B)	33
Yogurt	Plain yogurt (A)	8	Full-fat Stracciatella yogurt (C)	21	Fruit yogurt (B)	34
Potato	Boiled potato (A)	9	Paprika flavored chips (D)	22	Oven-baked fries (B)	35
Pasta	Plain cooked fusilli (A)	10	Ready-to-eat pasta ham and cheese (C)	23	Ready-to-eat pasta pesto with vegetables (B)	36
Tomato	Canned tomato (A)	11	Tomato ketchup (C)	24	Reduced sugar and salt ketchup (B)	37
Cheese	Young matured cheese 30+ (D)	12	Cream cheese with herbs (E)	25	Cottage cheese with sweet chili flavor (B)	38
Nuts	Unsalted peanuts (B)	13	Chocolate peanuts (D)	26	Oatmeal bar (B)	39

No* refers to the product number used in results section: MPF, minimally processed food (NOVA 1 or 3); UPF, ultraprocessed food (NOVA 4).

**Table 2:** Sociodemographic characteristics of Dutch, Brazilian, and Italian consumers.

Consumer Characteristic	Netherlands *n* = 277 % (*n*) *	Italy *n* = 204 % (*n*)	Brazil *n* = 181 % (*n*)
Age, years			
18–25	33 (90)	30 (61)	12 (22)
26–35	19 (53)	14 (29)	29 (52)
36–45	16 (44)	16 (33)	36 (65)
46–55	21 (58)	27 (55)	9 (16)
56–65	12 (32)	13 (26)	14 (26)
Male	33 (90)	22 (42)	37 (67)
Educational level			
Low	2 (4)	10 (19)	0 (0)
Moderate	20 (55)	41 (79)	18 (32)
High	77 (214)	49 (94)	82 (144)
Income level			
Student	18 (49)	17 (33)	7 (13)
Low	27 (73)	16 (30)	3 (5)
Moderate	14 (39)	48 (92)	43 (76)
High	32 (88)	7 (14)	34 (60)
Prefer not to answer	24 (9)	12 (23)	13 (22)

* All values are presented as percentage of consumers (absolute number of consumers).

## Data Availability

Not applicable.

## Supplementary Materials

The following supporting information can be downloaded at: https://www.mdpi.com/article/10.3390/nu14204438/s1, Table S1. Selected product categories with product pictures divided by degree of processing and healthiness, Table S2. Medians (IQR) of healthiness perception (1” not healthy at all”. 7 “very healthy”) of various food items in 13 categories of Dutch (*n* = 104–108), Italian (*n* = 75–83) and Brazilian (65–74) consumers, Table S3. Medians ((IQR) of processing perception (1” not processed at all”. 7 “very processed”.) of various food items in 13 categories of Dutch (*n* = 104–108), Italian (*n* = 75–83) and Brazilian (65–74) consumers.
