# Supplementary material for: Consumer Awareness of the Degree of Industrial Food Processing and the Association with Healthiness—A Pilot Study
Source: Nutrients. 2022 Oct 21;14(20):4438. doi: 10.3390/nu14204438 (PMC9610034; doi:10.3390/nu14204438)
Supplement: Supplementary file 1 [file nutrients-14-04438-s001.zip › nutrients-1973737-supplementary.pdf]

nutrients

MDPI

Supplementary Table S1. Selected product categories with product pictures divided by degree of processing and healthiness.

Products categories		Food products: Minimally processed/processed foods (NOVA 1/NOVA 3)	Food products: Ultra–Processed foods (NOVA 4) Unhealthy (Nutri–Score C, D or E)	Food products: Ultra–Processed foods (NOVA 4) Healthy (Nutri–Score A or B)
**Cereals and derivatives**	Cereals	Oatmeal (NOVA 1, Nutri–Score A) 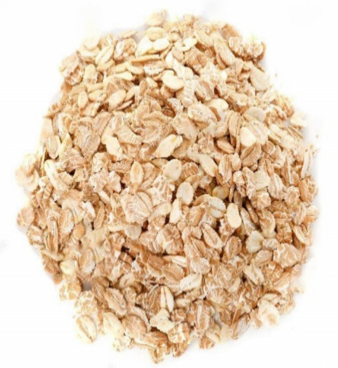	Cereals filled with chocolate (Nutri–Score D) 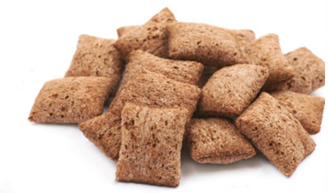	Granola with nuts, seeds and honey (Nutri–Score A) 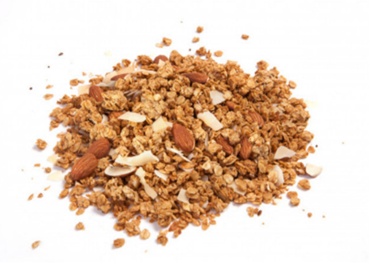
	Bread	Freshly baked French bread (NOVA 3, Nutri–Score B) 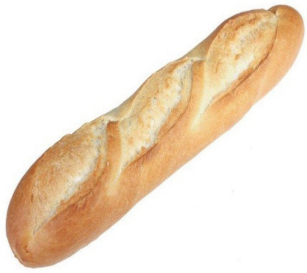	Freshly baked butter croissant. (Nutri–Score D) 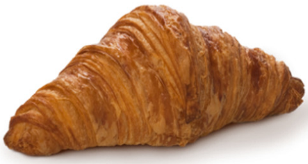	Packaged bread with multiple seeds (Nutri–Score A) 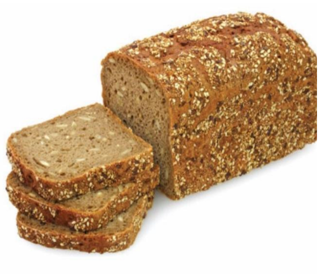
	Biscuits	Rice waffle with sea salt. (NOVA 3, Nutri–Score A)[1] 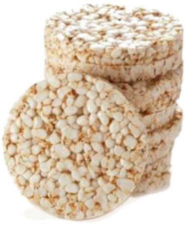	Wheat cookie. (Nutri–Score E) 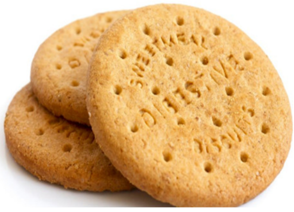	Grain cracker with multiple seeds. (Nutri–Score B) 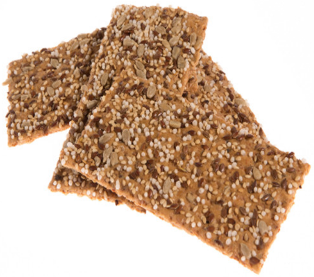

Sugar and sodas	Beverages	Freshly squeezed orange juice. (Nutri–Score B, NOVA 1) 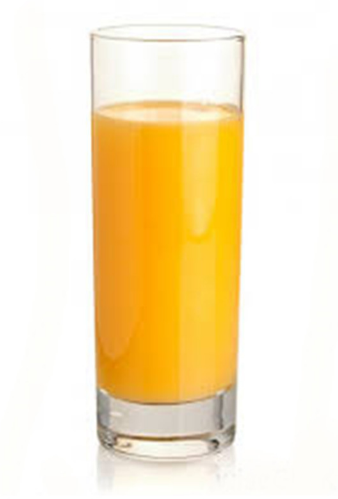	Sparkling lemonade (Nutri–Score D) 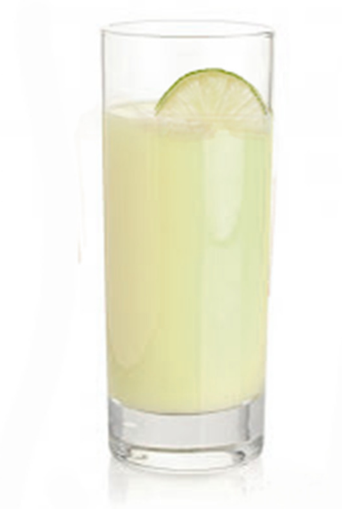	Cola light (Nutri–Score B) 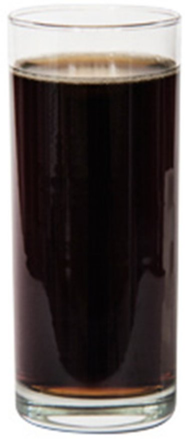
Meats	Beef	Medium rare steak (Nutri–Score A when uncooked when cooked depends on the amount of oil and salt used etc can become b or c then. NOVA 1) 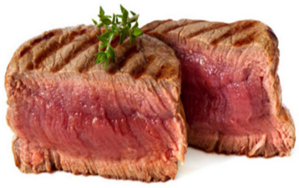	Sausage (Nutri–Score D) 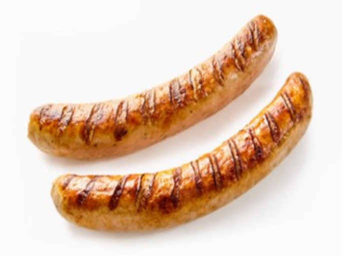	Beef replacer with vegetables (Nutri–Score A). 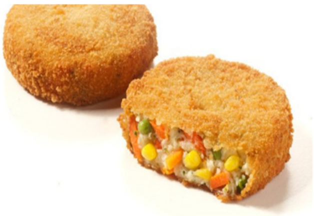
	Chicken	Roasted chicken filets. (NOVA 3, Nutri–Score A)[1] 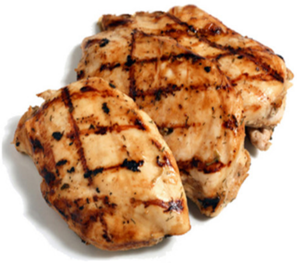	Fried chicken nuggets. (Nutri–Score D) 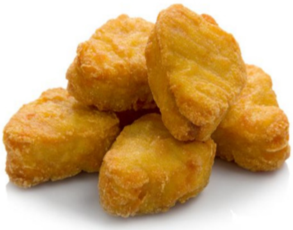	Tofu (Nutri–Score A) 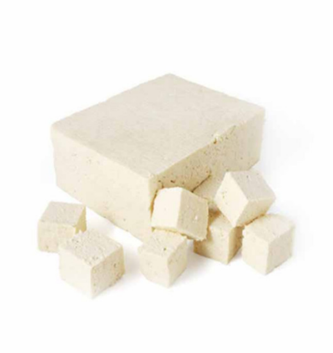
Milk and derivatives	Milk	Pasteurized semi skimmed milk (Nutri–Score A, NOVA 1) 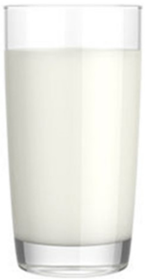	Skimmed milk flavoured with strawberry juice (Nutri–Score E) 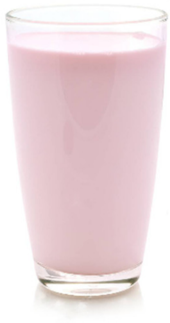	Semi–skimmed chocolate milk without added sugars (Nutri–Score B) 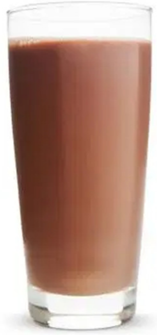

	Yogurt	Semi–skimmed plain yogurt (Nutri–Score A, NOVA 1)<br>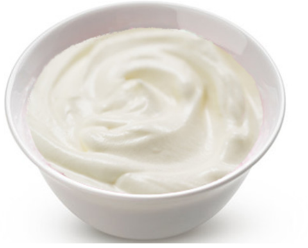	Stracciatella full–fat yogurt (Nutri–Score C)<br>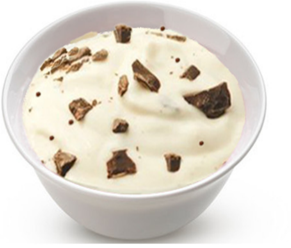	Semi–skimmed fruit flavoured yogurt (Nutri–Score B)<br>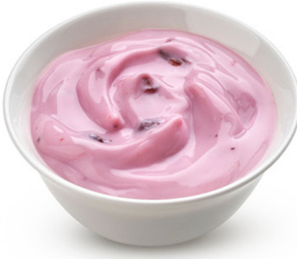
Potato		Boiled potato (Nutri–Score A, NOVA 1)<br>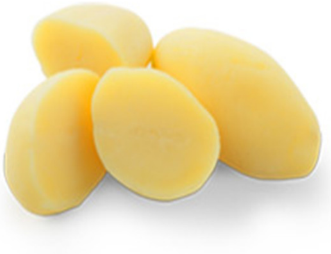	Paprika flavoured chips (Nutri–Score D)<br>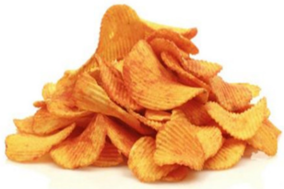	Oven baked French fries with no added salt (Nutri–Score B)<br>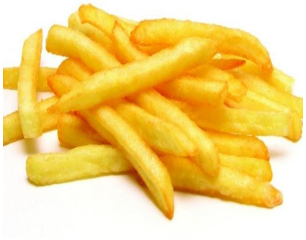
Ready–to–eat meals		Cooked fusilli pasta (Nutri–Score A, NOVA 1)<br>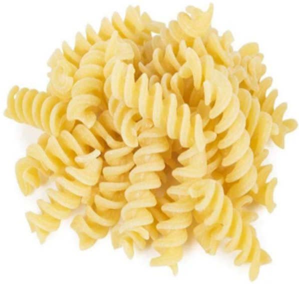	Ready to eat pasta with red sauce containing ham and cheese (Nutri–Score C)[1]<br>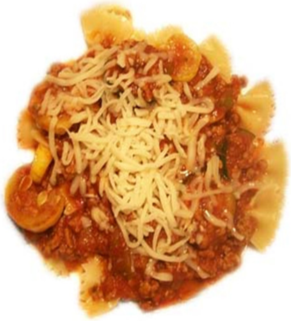	Ready to eat pasta pesto with vegetables (Nutri–Score B)<br>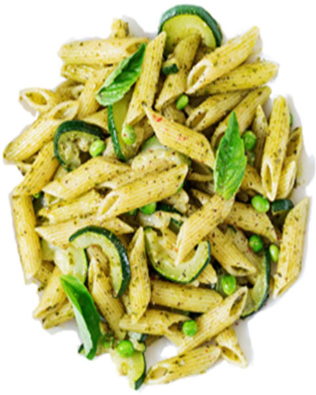
Vegetables	Tomato	Canned tomato (NOVA 3 Nutri–Score A)<br>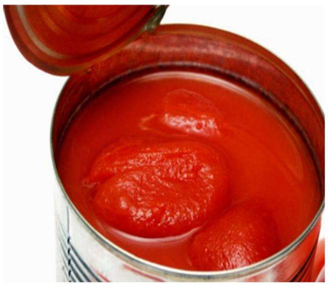	Tomato ketchup (Nutri–Score C)<br>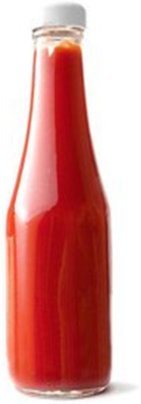	Tomato ketchup with reduced sugar and salt (Nutri–Score B)<br>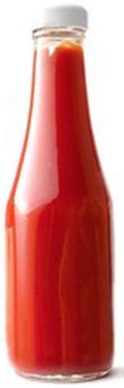

		Young matured cheese 30+ (Nutri–Score D NOVA 3) 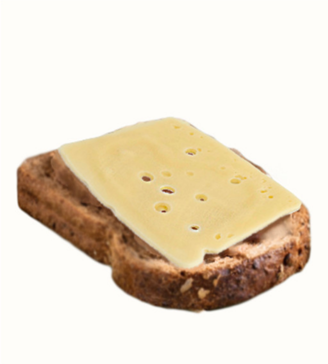	Cream cheese with herbs (Nutri–Score E) 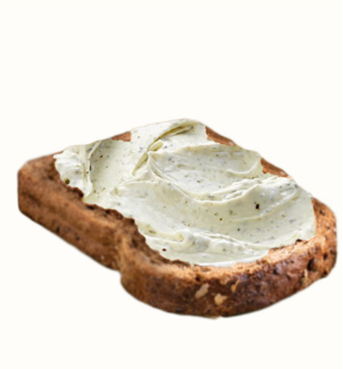	Cottage cheese with sweet chili flavour (NOVA 4, Nutri–score B) 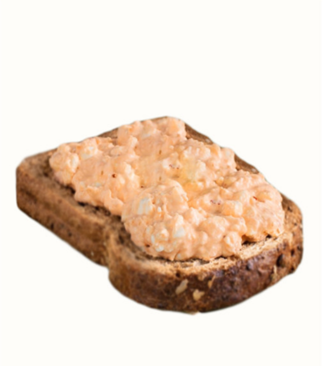
Others	Cheese			
		Unsalted peanuts (Nutri–Score B, NOVA 1) 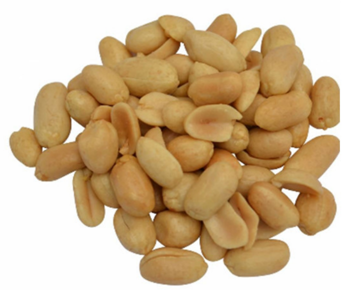	Milk chocolate peanuts (Nutri–Score D)[1] 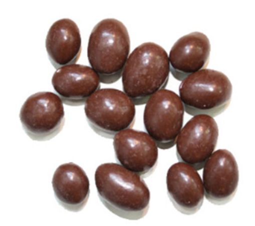	Oatmeal bar (Nutri–Score B) 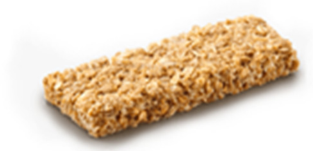
	Nuts			

1. Food pictures retrieved from the food–pics extended database [1,2].

Supplementary Table S2. Medians (IQR) of healthiness perception (1"not healthy at all". 7 "very healthy") of various food items in 13 categories of Dutch (n = 104–108), Italian (n = 75–83) and Brazilian (65–74) consumers.

Product Category[1]	Nationality	MPF	High NS_UPF	Low NS_UPF	*p*
Cereals	Dutch	6 (6–7)^{a}	5 (5–6)^{b}	2 (1–3)^{c}	<0.001
	Italian	6 (5–7)^{a}	6 (5– 7)^{a}	2 (1–3)^{b}	<0.001
	Brazilian	7 (6–7)^{a}	6 (5–7)^{b}	2 (1–3)^{c}	<0.001
	**All**	**7 (6–7)^{a}**	**6 (5–7)^{b}**	**2 (1–3)^{c}**	**<0.001**
Bread	Dutch	3 (2–5)^{a}	5 (5–6)^{b}	2 (1–3)^{c}	<0.001
	Italian	5 (4–6)^{a}	4 (3–5)^{b}	3 (2–3.5)^{c}	<0.001
	Brazilian	3 (2–5)^{a}	5 (4–6)^{b}	2 (1–3)^{c}	<0.001
	**All**	**4 (3–5)^{a}**	**5 (4–6)^{b}**	**2 (1–3)^{c}**	**<0.001**
Biscuits	Dutch	5 (4–6)^{a}	6 (5–6)^{b}	4 (3–5)^{c}	<0.001
	Italian	6 (4–7)^{a}	5 (5–6)^{a}	4 (3–5)^{b}	<0.001
	Brazilian	5 (4–6)^{a}	5 (3–6)^{a}	2 (1–4)^{b}	<0.001
	**All**	**5 (4–6)^{a}**	**5 (5–6)^{b}**	**3 (2–5)^{c}**	**<0.001**
Beverages	Dutch	6 (4–6)^{a}	2 (1–3)^{b}	2 (1–2)^{b}	<0.001
	Italian	7 (6–7)^{a}	1 (1–2)^{b}	3 (2–5)^{c}	<0.001
	Brazilian	7 (6–7)^{a}	1 (1–1)^{b}	4 (2–6)^{c}	<0.001
	**All**	**6 (5–7)^{a}**	**1 (1–2)^{b}**	**2 (1–4)^{c}**	**<0.001**
Meat/beef	Dutch	5 (3–6)^{a}	4 (3–5)^{b}	2 (1.5–3)^{c}	<0.001
	Italian	5 (4–6)^{a}	4 (2–5)^{b}	3 (2–3)^{c}	<0.001
	Brazilian	6 (5–7)^{a}	2.5 (1–5)^{b}	1 (1–2)^{c}	<0.001
	**All**	**5 (4–6)^{a}**	**4 (2–5)^{b}**	**2 (1–3)^{c}**	**<0.001**
Chicken	Dutch	5 (4–6)^{a}	5 (4–6)^{a}	1 (1–2)^{b}	<0.001
	Italian	6 (5–7)^{a}	5 (4–6)^{b}	1 (1–2)^{c}	<0.001

	Brazilian	7 (6–7)^{a}	6 (5–7)^{b}	1 (1–2)^{c}	<0.001
	**All**	**6 (5–7)^{a}**	**5 (4–6)^{b}**	**1 (1–2)^{c}**	**<0.001**
Milk	Dutch	5 (5–6)^{a}	3 (2–4)^{b}	3 (2–5)^{b}	<0.001
	Italian	5 (5–7)^{a}	3 (3–5)^{b}	3 (2–4)^{b}	<0.001
	Brazilian	5 (3–5)	3 (2–5)	3 (2–5)	0.063
	**All**	**5 (4–6)^{a}**	**3 (2–5)^{b}**	**3 (2–5)^{b}**	**<0.001**
Yogurt	Dutch	6 (5–6)^{a}	3 (3–5)^{b}	2 (2–3)^{c}	<0.001
	Italian	6 (5–7)^{a}	4 (3–6)^{b}	4 (3–5)^{b}	<0.001
	Brazilian	6 (5–7)^{a}	5 (4–6)^{b}	4 (3–6)^{b}	<0.001
	**All**	**6 (5–7)^{a}**	**4 (3–5)^{b}**	**3 (2–5)^{c}**	**<0.001**
Potatoes	Dutch	6 (5–6)^{a}	3 (2.5–5)^{b}	1 (1–2)^{c}	<0.001
	Italian	6 (5–7)^{a}	3 (2–4)^{b}	1 (1–2)^{c}	<0.001
	Brazilian	6 (5–7)^{a}	4 (2–5)^{b}	1 (1–2)^{c}	<0.001
	**All**	**6 (5–7)^{a}**	**3 (2–5)^{b}**	**1 (1–2)^{c}**	**<0.001**
Ready to eat pasta	Dutch	4 (3–5)^{a}	4 (3–5)^{b}	2 (1–2)^{c}	<0.001
	Italian	5 (4–6)^{a}	5 (3–6)^{b}	3 (1–4)^{c}	<0.001
	Brazilian	4 (3–5)^{a}	5 (3–6)^{a}	2 (1–3)^{b}	<0.001
	**All**	**5 (3–5)^{a}**	**4 (3–5)^{b}**	**2 (1–3)^{c}**	**<0.001**
Tomato	Dutch	5 (3–6)^{a}	4 (3–5)b^{b}	3 (2–4)^{c}	<0.001
	Italian	5 (4–6)^{a}	3 (2–4)^{b}	2 (1–3)^{c}	<0.001
	Brazilian	4 (2–5)^{a}	3 (1–3)^{b}	2 (1–3)^{c}	<0.001
	**All**	**5 (3–6)^{a}**	**3 (2–4)^{b}**	**2.5 (1–3)^{c}**	**<0.001**
Cheese	Dutch	5 (4–6)^{a}	3 (3–5)^{b}	3 (2–4)^{c}	<0.001
	Italian	4 (3–5)^{a}	3 (2–4.5)^{b}	3.5 (3–5)^{ab}	0.050
	Brazilian	5 (3–6)^{a}	4 (3–6)^{ab}	4 (2–5)^{b}	0.004
	**All**	**5 (3–5)^{a}**	**3 (2–5)^{b}**	**3 (2–5)^{b}**	**<0.001**
Peanuts	Dutch	6 (5–6)^{a}	4 (3–5)^{b}	2 (1–3)^{c}	<0.001
	Italian	4 (3–6)^{a}	5 (4–6)^{b}	2.5 (2–3)^{c}	<0.001
	Brazilian	6 (5–7)^{a}	5 (3–5)^{b}	2 (1–3)^{c}	<0.001
	**All**	**5 (4–6)^{a}**	**5 (3–5)^{b}**	**2 (1–3)^{c}**	**<0.001**

^{a,b,c,} Different letters indicate significant differences ($p < 0.05$) of post hoc comparisons. Bold numbers indicate the medians (IQR) and p-values of the countries together. MPF = minimally processed food; High NS_UPF = ultra-processed food with high Nutri_Score; Low NS_UPF= ultra-processed food with low Nutri-Score.

Supplementary Table S3. Medians (IQR) of processing perception (1″ not processed at all". 7 "very processed".) of various food items in 13 categories of Dutch (n = 104–108), Italian (n = 75–83) and Brazilian (65–74) consumers.

**Product category**[1]	**NOVA classification**	**MPF**	**High NS_UPF**	**Low NS_UPF**	***p***
Cereals	Dutch	2 (2–3)^{a}	4 (3–5)^{b}	6 (5–7)^{c}	<0.001
	Italian	3 (2–5)^{a}	3 (2–5)^{a}	6 (5–7)^{b}	<0.001
	Brazilian	2 (1–3.5)^{a}	3 (1–4)^{a}	6 (5–7)^{b}	<0.001
	**All**	**2 (2–4)^{a}**	**4 (2–5)^{b}**	**6 (5–7)^{c}**	**<0.001**
Bread	Dutch	5 (3–5)^{a}	5 (3–6)^{a}	5 (4–6)^{b}	0.021
	Italian	4 (3–5)^{a}	5 (4–6)^{b}	5 (4–6)^{b}	<0.001
	Brazilian	5 (3–6)	5 (3–6)	5 (3–7)	0.55
	**All**	**5 (3–5)^{a}**	**5 (4–6)^{b}**	**5 (4–6)^{b}**	**<0.001**
Biscuits	Dutch	4 (3–5)^{a}	5 (3–5)^{a}	5 (4–6)^{b}	<0.001
	Italian	5 (3–6)	5 (4–6)	5 (4–6)	0.21
	Brazilian	5 (3–6)^{a}	5 (3–6)^{a}	6 (5–7)^{b}	<0.001

	**All**	**5 (3–5.5)[a]**	**5 (3–5)[a]**	**5 (4–6)[b]**	**<0.001**
Beverages	Dutch	2 (1–3)[a]	7 (6–7)[b]	6 (5–7)[c]	<0.001
	Italian	2 (1–3)[a]	7 (6–7)[b]	5 (3–6)[c]	<0.001
	Brazilian	1 (1–2)[a]	7 (7–7)[b]	5 (3–6)[c]	<0.001
	**All**	**2 (1–3)[a]**	**7 (6–7)[b]**	**5 (4–7)[c]**	**<0.001**
Meat/beef	Dutch	3 (2–4)[a]	6 (5–6)[b]	5 (5–6)[b]	<0.001
	Italian	2 (1–3)[a]	6 (5–7)[b]	5 (4–7)[b]	<0.001
	Brazilian	2 (1–3)[a]	6 (5–7)[b]	7 (6–7)[c]	<0.001
	**All**	**2 (1–3)[a]**	**6 (5–7)[b]**	**6 (5–7)[b]**	**<0.001**
Chicken	Dutch	3 (2–5)[a]	4 (3–5)[b]	7 (6–7)[c]	<0.001
	Italian	3 (2–5)[a]	5 (3–6)[b]	6 (5–7)[c]	<0.001
	Brazilian	2 (1–3)[a]	3 (2–5)[b]	7 (6–7)[c]	<0.001
	**All**	**3 (2–5)[a]**	**4 (3–5)[b]**	**7 (6–7)[c]**	**<0.001**
Milk	Dutch	4 (2–5)[a]	5 (5–6)[b]	5 (5–6)[b]	<0.001
	Italian	5 (3–5)[a]	5 (4–6)[b]	5 (5–6)[b]	<0.001
	Brazilian	5 (3–6)[a]	6 (5–6)[b]	6 (5–7)[b]	0.004
	**All**	**4 (3–5)[a]**	**5 (5–6)[b]**	**5 (5–6)[b]**	**<0.001**
Yogurt	Dutch	3 (2–5)[a]	5 (5–6)[b]	6 (5–6)[b]	<0.001
	Italian	3.5 (2–5)[a]	5 (4–5)[b]	5 (3.5–5.5)[b]	<0.001
	Brazilian	4 (3–5)[a]	5 (5–6)[b]	5 (4–6)[b]	<0.001
	**All**	**4 (2–5)[a]**	**5 (5–6)[b]**	**5 (4–6)[b]**	**<0.001**
Potatoes	Dutch	2 (1–2)[a]	4 (3–5)[b]	6 (5–7)[c]	<0.001
	Italian	1 (1–2)[a]	4 (3–5)[b]	6 (6–7)[c]	<0.001
	Brazilian	1 (1–2)[a]	4 (2–5)[b]	7 (6–7)[c]	<0.001
	**All**	**1 (1–2)[a]**	**4 (3–5)[b]**	**6 (6–7)[c]**	**<0.001**
Ready to eat pasta	Dutch	4 (3–5)[a]	4 (3–5)[b]	6 (6–7)[c]	<0.001
	Italian	4 (3–5)[a]	5 (3–6)[b]	6 (5–7)[c]	<0.001
	Brazilian	5 (3–6)[a]	4 (3–5)[a]	6 (5–7)[b]	<0.001
	**All**	**4 (3–5)[a]**	**5 (4–6)[b]**	**6 (5–7)[c]**	**<0.001**
Tomato	Dutch	5 (3–6)[a]	5 (5–6)[b]	5 (5–6)[b]	<0.001
	Italian	4 (3–5)[a]	5 (4.5–6.5)[b]	6 (5–7)[b]	<0.001
	Brazilian	5 (3–7)[a]	6 (5–7)[b]	6 (5–7)[b]	<0.001
	**All**	**5 (3–6)[a]**	**6 (5–7)[b]**	**6 (5–7)[b]**	**<0.001**
Cheese	Dutch	4 (3–5)[b]	5 (5–6)[a]	5 (5–6)[a]	<0.001
	Italian	5 (4–6)	5 (4–6)	5 (4–6)	0.048
	Brazilian	5 (3–6)[a]	5 (4–6)[ab]	5 (5–6)[b]	0.040
	**All**	**5 (3–5)[a]**	**5 (4–6)[b]**	**5 (5–6)[b]**	**<0.001**
Peanuts	Dutch	2 (1–3)[a]	5 (5–6)[b]	5 (5–6)[b]	<0.001
	Italian	3 (2–4)[a]	5 (4–6)[b]	5 (5–6)[c]	<0.001
	Brazilian	2 (1–3)[a]	5 (3–7)[b]	5 (4–6)[b]	<0.001
	**All**	**2 (1–4)[a]**	**5 (4–6)[b]**	**5 (5–6)[b]**	**<0.001**

[a,b,c] Different letters indicate significant differences ($p < 0.05$) of post hoc comparisons. Bold numbers indicate the medians (IQR) and p-values of the countries together. MPF = minimally processed food; High NS_UPF = ultra-processed food with high Nutri_Score; Low NS_UPF= ultra-processed food with low Nutri-Score.

References

1. Blechert, J.; Lender, A.; Polk, S.; Busch, N.A.; Ohla, K. Food-pics_extended—An image database for experimental research on eating and appetite: Additional images, normative ratings and an updated review. *Front. Psychol.* **2019**, *10*, 307. https://doi.org/10.3389/fpsyg.2019.00307.

2. Blechert, J.; Meule, A.; Busch, N.A.; Ohla, K. Food-pics: An image database for experimental research on eating and appetite. *Front. Psychol.* **2014**, *5*, 617. https://doi.org/10.3389/fpsyg.2014.00617.
